# 

**Published:** 1904-12-03

**Authors:** 


NOTICE TO CORRESPONDENTS.

All MSS., Letters, Books for Review, and other matters intended for the Editor should be addressed The Editor, "The Hospital," The Hospital Buildings, 28 & 29 Southampton Street, Strand, W.C.

The Editor cannot undertake to return rejected MS., even when accompanied by stamped directed envelope.

**Notice to Advertisers and Subscribers.**

All Advertisements, Orders for Copies of The Hospital, and Business Communications should be addressed to **THE MANAGER (Not to the Editor),** The Hospital, 28 & 29 Southampton Street, Strand, London, W.C.

The Cost of Subscription, post free, is as follows:—Per week, 3½d.; per quarter, 3s. 3d.; per half-year, 6s. 6d.; per annum, 13s. Foreign and Colonial:—Per annum, 19s. 6d., payable, in all cases, in advance.

**NOTICE.—Vol. XXXVI. of "The Hospital" (April to September 1904), in handsome cloth binding, is now on sale at the office of this Journal, price 10s. 6d. Cases for binding the Numbers contained in this Volume 2s. Index to Vol. XXXVI., 3½d., post free.**

**The Monthly part for December is now ready. Price 1s., by post, 1s. 3d.**

### MEDICAL APPOINTMENTS.

**Arthur Edwin Boycott**, M.B., B.Ch.Oxon, Assistant Bacteriologist at the Lister Institute of Preventive Medicine. **R. G. Brown**, M.B., B.S.Aberd., Resident Assistant Medical Officer, Lambeth Parish Infirmary. **J. C. Muir**, M.D.Cantab., B.C., Medical Superintendent of the St. George's Infirmary, Fulham Road. **Jules F. Rey**, M.R.C.S., L.R.C.P.Lond., L D.S. Eng., House-Surgeon to the Royal Boscombe and West Hants Hospital. **F. W. B. Sugden**, District Medical Officer of the Hemsworth Union. **H. E. Symes-Thompson**, M.A., M.D.Cantab., M.R.C.P.Lond., Assistant Physician of the Royal Hospital for Diseases of the Chest, City Road, E.C. **Wilson Tyson**, M.B., B.C.Camb., Surgeon to the Lowestoft Hospital.

**"The Hospital" Institutional Library.**

**"Hospitals and Asylums of the World." 4 Vols., with a Portfolio of Plans, complete £12 12s.**

**"Burdett's Hospitals and Charities, 1904." 5s. net.**

**"Burdett's Official Nursing Directory." 8s. net.**

**"Cottage Hospitals, General, Fever, and Convalescent." 10s. 6d.**

**"Uniform System of Accounts for Hospitals and Public Institutions." New Edition. 4s. net.**

**"Hospital Expenditure: The Commissariat." 2s. 6d.**

**"A Legal Handbook for the Use of Hospital Authorities." 2s. 6d.**

**"The Cottage Hospital Case Book or Register of Patients." 10s. and 12s. 6d.**

**All these are published by the SCIENTIFIC PRESS, Ltd., and may be obtained through any bookseller, or direct from the publishers, 28 and 29 Southampton Street, Strand, London, W.C.**

### Medical Appointments.

THE HOSPITAL FOR SICK CHILDREN, GREAT ORMOND STREET, LONDON, W.C.

Notice is hereby given that the office of RESIDENT MEDICAL SUPERINTENDENT will become vacant on December 18th, 1904.

Candidates for this post are invited to send in their applications addressed to the Secretary, with copies of not more than three testimonials given specially for the purpose, on or before 12 o'clock on WEDNESDAY, December 7th, 1904.

Candidates must be registered medical practitioners, and must have held a responsible hospital appointment.

The appointment is made for one year, but may be held, subject to annual re-election, for a period of three years.

Salary 100 guineas per annum, with Board and Residence in the hospital, and £5 washing allowance.

All candidates must be present and appear before the Joint Committees at their meeting on Thursday, December 8th, 1904, at 5 P.M. precisely.

Forms of application and copy of rules may be obtained from the Secretary.

By order of the Managing Committee,
JAMES McKAY, Acting Secretary.

November 8th, 1904. (2112)

### If You are a Nurse This Page will Interest You.

**A HANDBOOK FOR NURSES.**
By J. K. Watson, M.D., M.B., C.M.
New and Revised Edition. Profusely illustrated. **5s.** net, **5s. 4d.** post free.

**NURSING: Its Theory and Practice.**
Being a complete Text-book of Medical, Surgical, and Monthly Nursing.
By Percy G. Lewis, M.D., M.R.C.S., L.S.A.
New Edition. Profusely illustrated.
**3s. 6d.** post free.

**SURGICAL WARD-WORK AND NURSING.**
By Alexander Miles, M.D.Edin., C.M., F.R.C.S E.
Second Edition. With particulars and illustrations of the instruments used in various operations.
**3s. 6d.** net, **3s. 10d.** post free.

**OPHTHALMIC NURSING.**
By Sydney Stephenson, M.B., F.R.C.S.E.
New and Revised Edition.
**3s. 6d.** net, **3s. 10d.** post free.

**ELEMENTARY ANATOMY AND SURGERY FOR NURSES.**
By William McAdam Eccles, M.S.Lond., M.B., F.R.C.S., L.R.C.P.
**2s. 6d.** post free.

**ELEMENTARY PHYSIOLOGY FOR NURSES.**
By C. F. Marshall, M.D., B.Sc., F.R.C.S.
A valuable help to Physiology for Probationers.
Crown 8vo. illustrated, cloth.
**2s.** post free.

**NURSING IN DISEASES OF THE THROAT, NOSE, AND EAR.**
By P. MacLeod Yearsley, F.R.C.S.Eng., M.R.C.S., L.R.C.P.Lond.
**2s. 6d.** post free.

**HOSPITAL SISTERS AND THEIR DUTIES.**
By Eva C. E. Lückes, Matron, London Hospital.
Third Edition.
**2s. 6d.** post free.

**OUTLINES OF INSANITY.**
A Popular Treatise on the Salient Features of Insanity.
By Francis H. Walmsley, M.D.
Popular Edition.
**1s. 6d.** post free.

**A COMPLETE HANDBOOK OF MIDWIFERY.**
The Latest and Best Text-Book for the L.O.S.
By J. K. Watson, M.D.Edin.
143 Illustrations.
**6s.** net, **6s. 4d.** post free.

**A PRACTICAL HANDBOOK OF MIDWIFERY.**
By Francis W. Haultain, M.D.
Illustrated.
**6/-** net, **6/3** post free.

**PRACTICAL GUIDE TO SURGICAL BANDAGING AND DRESSINGS.**
By Wm. Johnson Smith, F.R.C.S.
A handy and useful reference book.
Suitable size for apron pocket.
**2s.** post free.

**THE EXAMINATION OF THE URINE.**
Compiled for the Use of Nurses.
By J. K. Watson, M.D., M.B., C.M.
**1s.** net, **1s. 1d.** post free.

**ART OF FEEDING THE INVALID.**
By a Medical Practitioner and a Lady Professor of Cookery.
New and Popular Edition.
**1/6** post free.

**THE NURSE'S PRONOUNCING DICTIONARY OF MEDICAL TERMS AND NURSING TREATMENT.**
By Honnor Morten.
New Edition, with phonetic pronunciations.
Edited by Mary I. Burdett.
**2/-** post free.

**THE HUMAN BODY.**
Its Personal Hygiene and Practical Physiology.
By B. P. Colton.
Profusely illustrated. **5s.** post free.

**MASSAGE AND MEDICAL GYMNASTICS, including the Swedish and Schott (NAUHEIM) Movements.**
By Axel V. Grafstrom, B.Sc., M.D.
**2s. 6d.** post free.

**ART OF MASSAGE.**
By Mrs. Creighton Hale.
Second Edition. **6s.** post free.

**THE SCIENTIFIC PRESS, LTD.**

Publishers of Nursing Manuals, Case-Books, and Charts,

28 & 29 Southampton St., Strand, London, W.C.

SEND POSTCARD FOR NEW CATALOGUE OF NURSING MANUALS.

